# Comparing Stakeholders’ Perspectives on Parkinson Disease Management and Digital Technologies: Exploratory International Survey

**DOI:** 10.2196/90377

**Published:** 2026-05-20

**Authors:** Jamie Linnea Luckhaus, Anna Kharko, Charlotte Blease, Maria-Luisa Almarcha-Menargues, Natalia Del Campo, Sofia Balula Dias, Beatriz Alves, Björn H Falkenburger, Leontios Hadjileontiadis, Maria Hägglund, Sara Riggare, Therese Scott Duncan

**Affiliations:** 1 Department of Women’s and Children’s Health, Participatory eHealth and Health Data, Faculty of Medicine, Uppsala University, MTC-huset, Dag Hammarskjölds väg 14B, 1 tr, Uppsala, 75237, Sweden, 46 0729999381; 2 Centre for Primary Care and Health Services Research, University of Manchester, Manchester, United Kingdom; 3 Digital Psychiatry, Department of Psychiatry, Beth Israel Deaconess Medical Center, Harvard University, Boston, MA, United States; 4 Neurology Department, Movement Disorders Unit, Hospital Ruber Internacional, Madrid, Spain; 5 NeuroToul Center of Excellence in Neurodegeneration (COEN) of Toulouse, University Hospital of Toulouse, Toulouse, Occitanie, France; 6 Center of Interdisciplinary Study of Human Performance (CIPER), Faculdade de Motricidade Humana, University of Lisbon, Lisbon, Portugal; 7 Faculdade de Motricidade Humana, University of Lisbon, Lisbon, Portugal; 8 Department of Neurology, Faculty of Medicine and University Hospital Carl Gustav Carus, Technische Universität Dresden, Dresden, Saxony, Germany; 9 Electrical & Computer Engineering Department, Aristotle University of Thessaloniki, Athens, Central Macedonia, Greece; 10 Khalifa University of Science and Technology, Abu Dhabi, United Arab Emirates; 11 Uppsala University Hospital, Uppsala, Sweden; 12 Center for Disability Studies, Uppsala University, Uppsala, Sweden

**Keywords:** artificial intelligence, AI, Parkinson disease, stakeholder perspectives, patient perspectives, self-care, predictive AI

## Abstract

**Background:**

Parkinson disease (PD) is a progressive neurodegenerative disorder that poses complex challenges for persons with PD, informal caregivers, and health care professionals. With growing interest in digital and predictive artificial intelligence (AI) tools for disease management, understanding the needs and digital readiness of these stakeholder groups is crucial.

**Objective:**

This work aims to (1) identify digital practices for PD management among persons with PD, at-risk individuals, caregivers, and health care professionals; (2) compare these practices across groups; (3) explore stakeholder desires for AI-based tools; and (4) assess alignments and gaps to inform tailored AI solutions.

**Methods:**

An anonymous cross-sectional online survey of an exploratory nature was distributed (from December 2024 to October 2025) in 5 languages and completed by 255 respondents. Descriptive statistics summarized responses to 41 questions, including stakeholder-specific items. *χ*^2^ tests were performed to examine stakeholder differences in desired AI features.

**Results:**

Interest in predictive AI was high across stakeholder groups. Symptom tracking was the most desired feature (selected by more than 76% of the respondents), and personalized treatment recommendations came second for both persons with PD and health care professionals; however, stakeholder priorities diverged in other areas. Health care professionals rated improving patient and informal caregiver engagement as significantly more important than persons with PD did, *χ*^2^_1_ (n=205)=34.78, *P*<.001, and Cramer V=0.41. Despite considerable interest, the reported use of digital tools was limited, as most persons with PD did not use symptom-tracking apps or wearables, nor were they currently monitoring their condition, although many expressed intentions to begin.

**Conclusions:**

While predictive AI tools were viewed positively across groups, there were significant gaps in stakeholder preferences, highlighting the importance of tailored, context-aware design. Early diagnosis was not prioritized by persons with PD or health care professionals, likely reflecting the complexity of diagnosing PD in the absence of disease-modifying therapies. Coupled with the emphasis placed on preventive lifestyle guidance by persons with PD and those at risk, this highlights the importance of actionability in AI-based monitoring and prediction. Such actionability may also enhance perceived relevance and uptake, given that reported interest in digital health tools and self-tracking exceeded actual use. These findings offer early-stage insight to guide the development of future AI-based solutions for PD.

## Introduction

Parkinson disease (PD) is a complex and progressive neurodegenerative disorder that presents significant challenges in many aspects of life for people with PD themselves, informal caregivers such as spouses or other family members, as well as for health care providers (HCPs) [1-3]. The majority of PD cases are idiopathic (of unknown cause), though a small percentage are hereditary [4]. As PD progresses, symptoms often fluctuate and intensify over time, requiring people with PD and their informal caregivers to continually adapt and refine self-management strategies to meet changing needs.

It is predicted that 22.5 million people worldwide will be living with PD in 2050, with PD being the second most common neurodegenerative disorder [5]. In Europe, the prevalence and incidence rates of PD in 2020 were estimated at approximately 108 to 257 per 100,000 and 11 to 19 per 100,000 per year, respectively [6]. Parallel to the increase in PD cases is the expansion of digital health solutions. The global digital health care market reached US $180.2 billion in 2023 and is projected to grow to US $549.7 billion by 2028, with an estimated 25% compound annual growth rate [7]. A scoping review of patients’ perspectives on digital health tools found that empowerment, self-management, and personalization were among the strongest drivers of uptake and highlighted the importance of participatory design [8]. In line with these results, effective PD management increasingly emphasizes the importance of self-management, recognizing the active role patients play in their own health. Research highlights how patients actively draw on both internal resources (eg, knowledge, self-tracking, and intrinsic motivation) and external support (eg, social networks, digital tools, and HCPs) to navigate the demands of illness [9]. In this context, digital health tools, including those powered by artificial intelligence (AI), can support symptom tracking, medication adherence, patient education, and communication with care teams.

A scoping review on mobile health (mHealth) tools for PD management found that the majority of tools support clinical symptom assessment, but there is a need for more tools that facilitate self-care through symptom management [10]. Additionally, predictive AI is a novel and promising addition to PD management, though AI likely requires mobile apps and smartwatches to collect data, distinguishing it from traditional self-management systems, warranting further research. One such initiative is AI-PROGNOSIS [11], a European Union–funded project that aims to improve PD diagnosis, monitoring, and treatment through predictive AI. To ensure user-centered design, it is critical to understand the current experiences, expectations, and digital readiness of the diverse groups involved in PD care. However, despite growing interest in AI for PD, user priorities and perspectives on these tools remain unclear, especially across different stakeholders.

Building on the identified gaps in understanding stakeholder perspectives, this study pursues 4 interconnected aims: (1) to identify current digital practices for PD management among people with PD, persons at risk of PD, caregivers, and HCPs, (2) to compare similarities and differences in these practices across stakeholder groups, (3) to explore stakeholder-specific desires and expectations for AI-based tools, including predictive and preventive applications, and (4) to assess how these practices and preferences align or diverge, thereby highlighting unmet needs and opportunities to inform the tailored design of future AI-driven solutions for PD care.

## Methods

### Study Design and Setting

This study is reported in accordance with the STROBE (Strengthening the Reporting of Observational Studies in Epidemiology) checklist for cross-sectional studies [12] (see Checklist 1) and the CHERRIES (Checklist for Reporting Results of Internet E-Surveys) [13].

The survey was conducted within the scope of the EU-funded AI-PROGNOSIS project, which aims to advance PD diagnosis and care through novel predictive models combined with digital biomarkers from everyday devices [11]. The survey was exploratory in nature.

The online survey consisted of 21 to 23 questions, with the exact number depending on the respondent’s group, that is, people with PD, persons at risk of PD, caregivers, and HCPs. Some questions asked respondents to rate statements. Including the latter, the survey consisted of 48 items. The survey included 15 to 17 mandatory (the number of questions depended on the stakeholder group) closed single-choice and multiple-choice questions, 48 mandatory 5-point Likert-scale items (ranging from 1*=Strongly disagree* to 3=*Neutral* to 5=*Strongly agree*), and 6 optional free-text comments. The questions were divided into 5 different sections: general information (16‐18 questions), perceived potential (6 questions), perceptions and attitudes (5 questions), intention to use (2 questions), and additional feedback (1 question). The number of general information questions comprised 7 universal questions and 9 to 11 stakeholder-specific questions, depending on the respondent group. The survey was designed by adopting a polytheoretical framework as a fusion of theories, frameworks, and models [14] to better understand the acceptance of AI-based tools in PD, namely the Theory of Planned Behavior [15], Social Cognitive Theory [16], the Health Belief Model [17], and the Technology Acceptance Model [18]. In particular, the Theory of Planned Behavior contributes insights into how attitudes, perceived norms, and perceived control shape stakeholders’ intentions to adopt AI-based tools. The Social Cognitive Theory emphasizes the role of self-efficacy and observational learning, highlighting how confidence in using digital technologies and exposure to peer practices may influence uptake. The Health Belief Model adds a focus on perceived susceptibility, severity, benefits, and barriers, which is particularly relevant for people with PD and caregivers evaluating preventive or supportive AI applications. Finally, the Technology Acceptance Model provides a lens on perceived usefulness and ease of use, central to understanding the willingness of HCPs and people with PDs to integrate AI into daily management. Together, this polytheoretical approach enables a more nuanced exploration of digital practices and desires, ensuring that the study captures both behavioral drivers and technological determinants across diverse stakeholder groups.

Using the EUSurvey [19] platform, the survey was available in English, French, German, Spanish, and Swedish. Translation was done in accordance with the World Health Organization [20], where a native speaker translated it and another native speaker back-translated the translation for cross-reference. Before dissemination, the survey design was pilot-tested by JLL through cognitive interviews with 2 persons with PD and 1 caregiver, which resulted in an expanded information text for each part.

### Data Collection

In this paper, only Part 1: General information (41 items between all stakeholder versions) was analyzed [21] (Multimedia Appendix 1), which was intended to provide an exploratory snapshot of digital health practices across stakeholders. Other survey sections address more detailed user preferences and require a larger sample size for robust analysis; these data are planned to be presented in a subsequent publication.

Survey dissemination was done through the clinical and patient networks of the AI-PROGNOSIS project partners, focusing on European countries where the research team’s available languages are spoken (the United Kingdom, France, Germany, Spain, and Sweden). However, no geographical restrictions were imposed, allowing responses from all countries. Data were collected between December 2024 and October 2025.

A nonprobability sampling method was used as an exploratory sample with no random selection. Inclusion criteria for respondents were being 18 years or older and self-identifying as one of the following: (1) having a Parkinson diagnosis, (2) being at risk of PD, (3) being related to someone with PD, (4) being a caregiver to a person with PD, or (5) a HCP currently working with persons with PD. Individuals who indicated that they are at risk of developing PD and individuals who indicated that they are related to someone with PD were combined to form the “at risk” group in the analysis.

### Analysis

We analyzed 41 questions (Part 1) from the AI-PROGNOSIS survey on a predictive AI-based tool for PD care [21] (Multimedia Appendix 1). These multiple-choice questions were analyzed descriptively and summarized through frequency tables, figures, and in-text descriptions. Similar responses (eg, “good” and “excellent”) were dichotomized where applicable. The analysis was carried out by JLL in JASP v0.19.1.0. Where the same question was asked of multiple stakeholder groups, these responses are presented side-by-side in figures or text for comparison. *χ*^2^ tests were performed to examine stakeholder differences in desired AI features. Because all comparisons involved 2×2 contingency tables, continuity correction by Yates was applied [22]. Open-ended qualitative questions were not included due to low response rates.

### Ethical Considerations

The survey responses were anonymous. The project was submitted for ethical review and was deemed exempt from formal approval by the Swedish Ethical Review Authority, Dnr 2024-05505-01.

## Results

### Overview

This paper presents findings from a survey on perspectives of AI and digital tools for Parkinson management across 4 stakeholder groups: people with PD, people at risk of developing PD, caregivers, and HCPs. Where relevant, findings are presented by the respondent group to reflect differing roles, experiences, and needs. As the respondent groups differ in sample size, the respondents (n) are presented in a table or in text (xx/xx, %).

### Respondent Characteristics

### Overview

A total of 255 responses were received from 18 different countries, with Spain (n=94, 37%) and France (n=66, 26%) contributing the most (Multimedia Appendix 2). Half (132/255, 51.8%) of the respondents were persons with PD (Table 1). Most of the persons with PD (67/132, 50.8%) and caregivers (8/21, 38.1%) were retired, while the majority of at-risk respondents (20/29, 69%) and HCPs (61/73, 83.6%) were employed full-time. Additionally, 79.2% (202/255) of all respondents had completed higher education (bachelor’s degree or above).

**Table 1.** Sociodemographic characteristics. Apart from the heading, percentages were calculated based on the column total, that is, per respondent group. The largest percentage for each group is indicated in italics.

Sociodemographic characteristics	People with Parkinson disease (n=132, 51.8%)	At risk (n=29, 11.4%)	Caregivers (n=21, 8.2%)	HCPs^a^ (n=73, 28.6%)	Whole sample (N=255, 100%)
Gender, n (%)					
Female	62 (47)	22 (75.9)	15 (71.4)	48 (65.8)	154 (60.4)
Male	69 (52.3)	7 (24.1)	6 (28.6)	24 (32.9)	99 (38.8)
Other	—^b^	—	—	—	—
Rather not say	1 (1)	—	—	1 (1.4)	2 (0.8)
Age (y), n (%)					
18‐24	—	—	—	3 (4.1)	3 (1.1)
25‐34	—	4 (13.8)	—	25 (34.2)	29 (11.4)
35‐44	7 (5.3)	5 (17.2)	1 (4.8)	24 (32.9)	37 (14.5)
45‐54	25 (18.9)	7 (24.1)	3 (14.3)	9 (12.3)	44 (17.3)
55‐64	37 (28)	10 (34.5)	8 (38.1)	11 (15.1)	66 (25.9)
65‐74	53 (40.2)	3 (10.3)	7 (33.3)	1 (1.4)	64 (25.1)
>75	10 (7.6)	—	2 (9.5)	—	12 (4.7)
Highest level of education, n (%)					
Primary school	2 (1.5)	—	—	—	2 (0.8)
Secondary/high school	19 (14.4)	1 (3.45)	3 (14.3)	1 (1.3)	24 (9.4)
Vocational education	20 (15.2)	—	4 (19)	3 (4.1)	27 (10.6)
Bachelor’s degree	41 (31.1)	14 (48.3)	8 (38.1)	22 (30.2)	85 (33.3)
Master’s degree	35 (26.5)	11 (37.9)	3 (14.3)	26 (35.6)	75 (29.4)
PhD or similar	15 (11.4)	3 (10.4)	3 (14.3)	21 (28.8)	42 (16.5)
Employment status, n (%)					
Employed full-time	31 (23.5)	20 (69)	6 (28.6)	61 (83.6)	118 (46.3)
Employed part-time	10 (7.6)	2 (6.9)	2 (9.5)	12 (16.4)	26 (10.2)
Retired	67 (50.8)	5 (17.1)	8 (38.1)	—	80 (31.3)
Unemployed	2 (1.5)	1 (3.5)	2 (9.5)	—	5 (2)
Disability allowance	10 (7.6)	1 (3.5)	1 (4.8)	—	12 (4.7)
Other	12 (9)	—	2 (9.5)	—	14 (5.5)

a HCPs: health care providers.

b Not applicable.

Most people with PD (53/132, 40.2%) were aged 65 to 74 years, and half were retired (67/132, 50.8%). People with PD reported a complex symptom profile, commonly citing bradykinesia (85/132, 64.4%) and rigidity (85/132, 64.4%) as the symptoms they experience most frequently and managing their PD with medication (123/132, 93.2%) and exercise (104/132, 78.8%). Nearly one-third were diagnosed 1 to 3 years ago (42/132, 31.8%), followed by 4 to 6 years ago (37/132, 28%). Individuals at risk consisted of 2 out of 29 (6.9%) participants who self-reported risk, and a majority of individuals were related to people with PD (classified as at-risk in this study). This group was generally younger than people with PD (10/29, 34.5% were 55‐64 years old) and employed (20/29, 69% full-time).

Caregivers were primarily spouses (12/21, 57.1%) or adult children (8/21, 36.1%), of which the majority had been providing care for over a decade (9/21, 42.9%) or 6 to 10 years (5/21, 23.8%). Their perceived support levels were notably low, with most rating the support they received as “very poor/poor” (14/21, 66.7%). The most positive support level reported was “fair” (6/21, 28.6%).

HCPs were typically younger, with most aged 25 to 44 (49/73, 67.1%) years and working full-time (61/73, 83.6%) in a hospital (45/73, 61.6%). The sample was professionally diverse, including neurologists, nurses, and physiotherapists.

### Experiences With Managing PD and Risk

The most reported health goals, according to people with PD, were to “minimize symptoms with lifestyle factors (eg, diet, physical activity, sleep habits)” (123/132, 93.2%) and “slow Parkinson disease progression through lifestyle factors (eg, diet, physical activity)” (112/132, 84.6%). The most commonly reported challenge among persons with PD was managing motor symptoms (70/132, 53%; Table 2).

**Table 2.** Greatest challenges in managing Parkinson disease according to people with Parkinson disease (N=132)^a^.

Challenges	n (%)
Managing motor symptoms	70 (53)
Managing nonmotor symptoms	17 (12.9)
Emotional or psychological impact	25 (18.9)
Lack of support or resources	5 (3.8)
Adhering to treatment plans	6 (4.5)
Accessing health care services	2 (1.5)
Financial burden	2 (1.5)
Other	5 (3.8)

a For multiple-choice questions, percentages may not add up to 100%.

Caregivers most often cited providing emotional support (14/21, 66.7%), other (14/21, 66.7%), balancing caregiving with other responsibilities (13/21, 61.9%), and helping with mobility and physical activities (12/21, 57.1%) as their primary caregiving challenges (Table 3). When asked about their knowledge of PD, nearly half of them rated it as “moderate” (10/21, 47.6%), and 43.9% (9/21) reported it as “high/very high.” The most frequently reported information sources were health care professionals (11/21, 52.4%) and educational websites (10/21, 47.6%).

**Table 3.** Greatest challenges in managing Parkinson disease according to caregivers (N=21)^a^.

Challenges	n (%)
Helping with mobility and physical activities	12 (57.1)
Providing emotional support	14 (66.7)
Managing symptoms (eg, tremor, rigidity)	9 (42.9)
Managing medication schedules	7 (33.3)
Coordinating with health care providers	6 (28.6)
Balancing caregiving with other responsibilities	13 (61.9)
Other	14 (66.7)

a For single-choice questions, percentages may not add up to 100%.

Like people with PD, HCPs also identified symptom management as a primary challenge in PD care. However, there was no difference in ranking of nonmotor symptoms (49/73, 67.1%) and motor symptoms (47/73, 64.4%), which was followed by “providing patient and caregiver education” (39/73, 53.4%; Table 4). When asked about the overall effectiveness of current treatments, half (36/73, 49.4%) of HCPs rated them as “good” or “excellent”, while 45.2% (33/73) rated them as “fair.” Regarding methods of caregiver involvement, HCPs most commonly reported providing education and resources (51/73, 69.9%), maintaining regular communication (46/73, 63%), and involving caregivers in treatment planning (43/73, 58.9%). Fewer reported offering support groups or counseling (35/73, 48%).

**Table 4.** Greatest challenges in managing Parkinson disease according to health care providers (N=73)^a^.

Challenges	n (%)
Managing motor symptoms	47 (64.4)
Managing nonmotor symptoms	49 (67.4)
Providing patient and caregiver education	39 (53.4)
Patient adherence to treatment plans	36 (49.3)
Coordinating care among multiple providers	26 (35.6)
Diagnosing the disease	17 (23.3)

a For multiple-choice questions, percentages may not add up to 100%.

In terms of managing PD risk, few had consulted a HCP regarding their perceived risk (5/29, 17.2%) or taken steps to manage it (eg, specific lifestyle recommendations, regular check-ups, self-tracking; 8/29, 27.6%). Perceived risk factors were not only primarily genetic predisposition (23/29, 79.3%) but also included age (11/29, 37.9%), environmental exposures (11/29, 37.9%), head injuries (5/29, 17.2%), and others (6/29, 20.7%). The majority (18/29, 62.1%) had not experienced any symptoms associated with prodromal PD, and levels of concern were evenly split between moderate-to-high concern (14/29, 48.3%) and little-to-no concern (15/29, 51.7%).

### Digital Literacy and Interest

**Figure 1.**
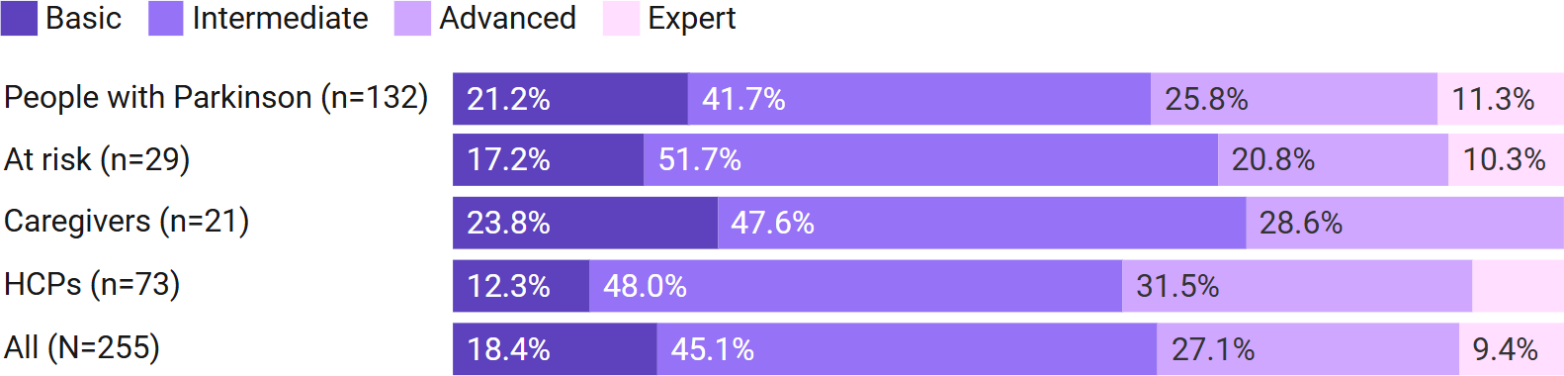
Self-reported digital literacy by stakeholder group (N=255). HCP: health care provider.

Across all groups, the majority self-rated their digital literacy level as intermediate (Figure 1). The lowest reported levels were among informal caregivers, although they were also the smallest sample.

Participants across all groups showed interest in using digital tools and AI to support PD (risk) management. The majority of at-risk respondents reported being “possibly” or “definitely” interested in using apps or wearable devices for monitoring risk or managing early symptoms (23/29, 79.3%). Similarly, most caregivers expressed interest in digital tools to support their caregiving responsibilities (14/21, 66.7%), and most HCPs reported interest in integrating AI into PD care (56/73, 76.7%).

Despite this interest, the current use of such tools remains limited. The majority of persons with PD reported not using a smartwatch or mobile tracking app (108/132, 81.8%). However, half expressed an intention to begin tracking (70/132, 53%), and nearly a quarter already did (31/132, 23.5%). No caregivers reported using mobile apps for PD care, and very few used wearables (3/21, 14.3%); most of them relied on observation and memory (18/21, 85.7%).

### Desired AI Features

**Figure 2.**
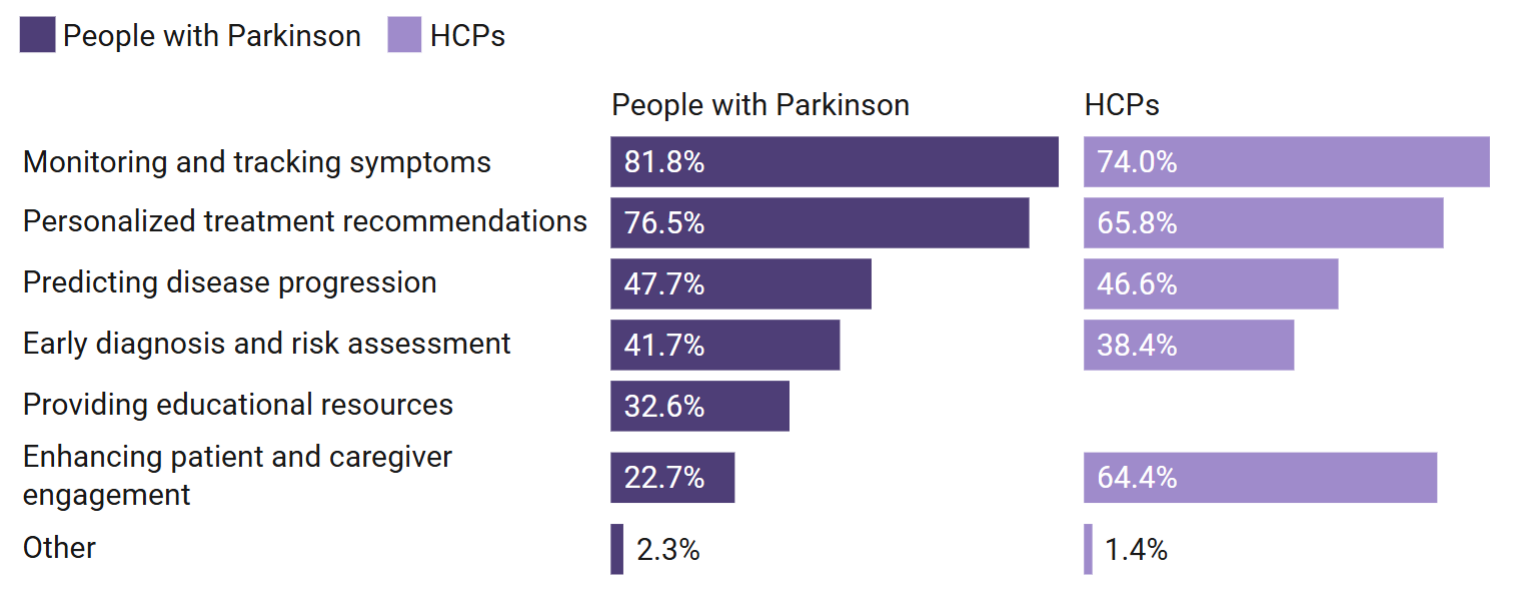
The most desired AI features according to people with Parkinson disease (n=132) and HCPs (n=73), shown side by side. Multiple-choice options; percentages may not add up to 100%. Values indicate the share of respondents who selected that feature. Question: “What features would you find most helpful in an AI-based tool for managing Parkinson disease? (Select all that apply).” “Providing educational resources” was only an option for people with Parkinson disease and not for HCPs. HCP: health care provider.

Respondents were presented with a stakeholder-specific list of potential AI features. The following paragraphs describe which features were prioritized within and across respondent groups (Figure 2). Symptom tracking emerged as the most frequently selected AI feature across all respondent groups, though secondary priorities varied between groups. HCPs also placed strong emphasis on tools that enhance patient and caregiver engagement (47/73, 64.4%), a priority less commonly noted by people with PD (30/132, 22.7%).

To further compare priorities for AI features between people with PD and HCPs, 5 *χ*^2^ tests of independence were conducted to determine whether these stakeholders select specific features at different rates. Group differences were not statistically significant for early diagnosis and risk assessment, *χ*²_1_ (n=205)=0.21, *P*=.64, Cramer V=0.03; monitoring and tracking symptoms, *χ*²_1_ (n=205)=1.75, *P*=.19, Cramer V=0.09; personalized treatment recommendations, *χ*²_1_ (n=205)=2.74, *P*=.10, Cramer V=0.12; or predicting disease progression, *χ*²_1_ (n=2o5)=0.03, *P*=.87, Cramer V=0.01. These effects were all small in magnitude.

A statistically significant and meaningful stakeholder difference was, however, found for enhancing patient and caregiver engagement, *χ*²_1_ (n=205)=34.78, *P*<.001, Cramer V=0.41. HCPs prioritized this feature significantly more frequently than people with PD, indicating a moderate-to-large association (Multimedia Appendix 3).

Preferences among individuals at risk for PD were examined separately using a tailored set of potential AI features. Among at-risk individuals (Table 5), AI-generated lifestyle recommendations aimed at prevention were more popular than progress reports and health insights (23/29, 79.3% and 15/29, 51.7%, respectively). Communication with health care professionals was the second most cited feature for both persons at risk (18/29, 62.1%) and caregivers (13/21, 61.9%; Table 6).

**Table 5.** Most desired artificial intelligence features according to people at risk of Parkinson disease (N=29)^a^.

Desired artificial intelligence features	n (%)
Symptom tracking	23 (79.3)
Medication reminders	4 (13.8)
Progress reports and health insights	15 (51.7)
Lifestyle recommendations (eg, diet, exercise)	23 (79.3)
Communication with health care providers	18 (62.1)
Community support and resources	12 (41.4)
Other	0 (0)

a Multiple-choice options; percentages will not add up to 100%. Values indicate the share of respondents who selected that feature. Question: “What features would you find most helpful in an AI-based tool for managing the risk of Parkinson’s disease? (Select all that apply).”

**Table 6.** Most desired artificial intelligence features according to caregivers (N=21)^a^.

Desired artificial intelligence features	n (%)
Symptom tracking	16 (76.2)
Medication reminders	8 (38.1)
Scheduling and appointment reminders	9 (42.9)
Educational resources	7 (33.3)
Communication with health care providers	13 (61.9)
Emergency alert features	6 (28.6)
Other	2 (9.5)

a Multiple-choice options; percentages will not add up to 100%. Values indicate the share of respondents who selected that feature. Question: “What features would you find most helpful in an AI-based tool for managing Parkinson’s disease? (Select all that apply).”

## Discussion

### Principal Findings

This study explored how different stakeholder groups—people with PD, people at risk, informal caregivers, and health care professionals—currently engage with digital tools, what they expect from AI-based solutions, and where their priorities converge or diverge in managing PD. The results highlight the complexity of managing PD and suggest both universal needs and distinct priorities across these 4 groups regarding the potential application of predictive AI. Interest in predictive AI was high across all stakeholder groups, with symptom tracking being the most desired feature; however, secondary priorities varied by group. Additionally, despite this interest, reported technological use was limited. Most of the people with PD did not use symptom-tracking apps or wearables, nor did they currently track their condition, though many expressed intentions to start. This pattern aligns with broader digital health literature demonstrating that interest in technology does not necessarily translate into adoption or sustained use [22]. Prior studies have found cost [23], discomfort (of wearables) [23], and fear of replacing in-person care [24] to constrain adoption for older adults with chronic illness. Addressing these barriers will be essential if AI-driven solutions are to move from intention to sustained practice.

### Comparing Stakeholder Groups

Based on this survey, the most critical insight for the development of new AI applications is the universal demand for symptom tracking; it was the most frequently selected desired AI feature across all respondent groups (ie, persons with PD, at-risk individuals, caregivers, and HCPs). This demand directly correlates with the perceived primary challenges in PD care, namely, for people with PD, the greatest challenge reported was managing motor symptoms; for HCPs, symptom management remains a primary challenge, with nearly equal emphasis on nonmotor symptoms and motor symptoms. Also, for caregivers, a symptom-tracking aid might be beneficial, given that they reported relying heavily on memory and observation for tracking. This consensus on symptom tracking supports the initiative to use digital biomarkers from everyday devices for PD monitoring, a direction in which many research projects are heading. Effective self-management, which is increasingly emphasized in PD care, depends heavily on using both internal resources (like self-tracking) and external support (like digital tools).

While symptom tracking was shared, the secondary priorities for AI tools reveal stakeholder-specific challenges that must be addressed to ensure user-centered design (Figure 2). In particular, at-risk respondents expressed strong interest in preventive lifestyle recommendations but reported limited engagement with HCPs regarding their risk. In contrast, the HCPs emphasized caregiver engagement and education above what people with PD did. These contrasts suggest the need for differentiated design approaches: preventive guidance for at-risk individuals, caregiver support features for HCPs and caregivers, and self-tracking tools for persons with PD.

### Demand for Actionable Insights

There is extensive evidence that many patients desire to take a more active role in their care and that digital health tools can aid in doing so. Additionally, research shows that people with PD and health care professionals desire to use digital health tools to facilitate co-care between them [25], and older adults have shown higher motivation to engage with technology when there is a clear value in terms of health outcomes [25,26].

This active role is tied closely to the primary health goals reported by people with PD in our study, which were centered on minimizing symptoms and slowing PD progression through lifestyle factors (eg, diet, physical activity, and sleep habits) or, in other words, factors that one can influence outside of health care. Similarly, individuals at risk, whose perceived risk factors include genetic predisposition and age, prioritized AI-generated lifestyle recommendations aimed at prevention. This shared focus confirms the importance of integrating nonpharmacological, preventative, and lifestyle-based recommendations into AI tools for both diagnosed individuals and those at risk.

A qualitative study within the AI-PROGNOSIS project also emphasized the demand for actionable insights, though it also demonstrated how individual and personal this question is. The degree to which participants felt they could influence their disease varied, as did their desire to know predictions about their disease [27]. This emphasis on actionability is consistent with prior work by Schaeffer et al [28], who found that expert clinicians and researchers in prodromal PD held divergent views on whether and when to disclose PD risk, with actionability emerging as a key factor in their reasoning. Together, these findings highlight the importance of individualized prognostic counseling for those at risk and with PD and the role of the clinician in determining the patient’s “wish to know” [28,29].

Stakeholder desires for AI tools extend beyond symptom monitoring to actionable, personalized insights [30,31]. In our study, people with PD emphasized lifestyle-based recommendations to minimize symptoms and slow progression, while at-risk individuals prioritized preventive guidance tailored to genetic and age-related risk. Caregivers highlighted the need for tools that reduce burden and provide structured support. The emphasis on actionable, personalized insights aligns with recent studies showing that the adoption of digital tools in PD is driven less by data collection itself and more by perceived clinical or lifestyle benefits [30,31]. These findings suggest that AI solutions should integrate predictive analytics with practical, user-centered recommendations that address both medical and nonmedical aspects of PD care.

### Communication and Engagement

HCPs desired tools that would enhance patient and caregiver engagement, and the literature demonstrated that HCPs recognize caregiver involvement as vital for the effective use of digital tools and clinical decision-making [32]. This priority directly addresses the third greatest challenge HCP respondents identified: providing patient and caregiver education. Respondents also reported involving caregivers by providing education and resources and maintaining regular communication. An AI tool could potentially aid in these tasks to overcome educational and communication gaps. The literature supports education as a challenge for HCPs, where a provider must balance how much to discuss and when. For example, discussing advanced PD services with someone having mild symptom presentation can cause unnecessary worry [33].

Notably, this desire for AI-facilitated engagement was less commonly cited by people with PD, who seemed to prioritize taking a more active role in self-managing their PD. However, family-centered care approaches in PD have been found to improve patient safety and self-efficacy [33]. A possible explanation for why people with PD did not prioritize patient and caregiver engagement to the extent HCPs did might be because patients who are able to participate in research may already be quite independent and less reliant on a caregiver and thus prioritize their own experience and features they find useful for self-care. Meanwhile, HCPs might focus on caregiver engagement due to the unique and clinically valuable insights a caregiver holds [28], especially among people with PD and cognitive symptoms. Research suggests that caregivers who utilize mHealth apps have substantially higher odds of engaging in health communication and making informed health decisions in collaboration with providers [33]. Consequently, HCPs are advised to encourage and support mHealth use among caregivers, which may be reflected in our results.

Communication with health care professionals was the second most cited desired feature for both caregivers and persons at risk. For caregivers, who listed emotional support and balancing responsibilities as main challenges, improving communication suggests a need for reliable channels to share information and seek guidance. For at-risk individuals, who rarely consult HCPs about their risk, AI-assisted monitoring may provide insights into when to connect with HCPs and provide data to help structure the visit.

### Balancing Prevention With Overdiagnosis

Early diagnosis was not a feature prioritized by people with PD or HCP respondents. Literature shows that diagnosing PD can profoundly affect mental health and identity, which may explain why some respondents may not welcome earlier detection in the absence of disease-modifying therapies and robust counseling [34]. Early-detection tools raise a prospective double-edged sword, as they could accelerate recruitment into trials and enable earlier supportive care, but without careful specificity, follow-up protocols, and shared decision-making, they may also widen harms from overdiagnosis. PD is commonly overdiagnosed, especially in its early stages and among nursing-home residents or patients assessed by clinicians without specialized training [35], and longitudinal evidence further indicates that initial PD diagnoses are frequently revised over time [36].

On the other hand, people with PD have typically already lost over half of their dopamine-producing neurons, so earlier diagnosis, in situations where such severe loss can be prevented, could be extremely valuable. For highly sedentary individuals, implementing regular high-intensity exercise before such extensive loss could potentially delay onset and symptom presentation. In a proof-of-concept study of patients with mild and early PD, 6 months of high-intensity exercise was found to induce beneficial neural changes, reversing the expected decline in key biomarkers of the dopaminergic system [37].

Taken together, the results reveal both convergence and divergence in stakeholder priorities. Convergence was evident in the universal demand for symptom tracking and interest in predictive AI. Divergence appeared in the emphasis on caregiver engagement among HCPs, preventive lifestyle guidance among at-risk individuals, and burden reduction among caregivers. Explicitly recognizing these shared and distinct needs is critical for participatory design, ensuring that future AI-driven solutions are broadly acceptable while remaining tailored to the unique contexts of each stakeholder group.

### Strengths and Limitations

This study used a nonprobability sampling approach, with recruitment primarily conducted through project networks and online dissemination channels. This strategy enabled participation from multiple countries and stakeholder groups, enhancing the overall diversity and breadth of perspectives captured. However, the same approach may have introduced sampling biases, including uneven representation across stakeholder groups and an overrepresentation of highly educated respondents, with 80% holding a bachelor’s degree or higher. Combined with the relatively small sample size and the exploratory nature of the study, these factors limit the generalizability of subgroup comparisons. Accordingly, the findings should be interpreted as preliminary and are intended to inform future large-scale studies using more systematic and inclusive recruitment strategies.

While multicountry inclusion is a strength, it also introduces challenges in interpretation. Contextual factors such as health care systems, access to digital tools, and cultural attitudes toward technology vary widely across the countries included. These wide contextual differences may have influenced how participants perceived and responded to questions about symptom management and digital support tools. Additionally, although the AI tools were briefly explained (including functions, features, and aims), differing understandings of AI likely influenced responses and acceptability.

### Future Directions

As AI-assisted tools become more of a reality than a fantasy, we must ensure robust user-centered design, which accounts for differences in stakeholder preferences. This entails participatory methods with efforts to include underrepresented populations, including those with lower education levels and participants outside of EU countries. As these AI-based tools develop, continued involvement of people with PD and stakeholders is crucial.

This survey identifies specific features stakeholders prioritize; future discussion should focus on how to overcome the practical obstacles in implementation and adoption to realize the potential of AI in PD care. Symptom tracking was a top desired feature, though there are already many tools available aimed at filling out this need. As suggested by Madanian et al [8], this is likely in part due to such tools being developed for HCPs rather than for patients or for collaboration between both parties. As HCPs emphasized engaging patients and caregivers, future research might examine how data collected and analyzed through AI tools (eg, detailed symptom tracking and prognosis) can be seamlessly integrated into clinical workflows in such a way as to facilitate shared decision-making.

Finally, building on the importance of participatory approaches in the design of AI-based solutions, the effective integration of AI into PD management depends on the development of tools that are trustworthy, inclusive, and transparent. Explainable and reliable AI systems are essential to safeguard patient safety and foster clinician confidence, while inclusive model design and validation help mitigate bias and promote equitable care across diverse populations. Transparency in AI-driven decision-making further supports ethical governance, facilitates clinical adoption, and enables informed use in real-world practice.

### Conclusion

This study aimed to identify and compare digital practices and desires for AI-based tools for PD among people with PD, persons at risk of PD, caregivers, and HCPs. The survey revealed high interest in AI-based tools for PD management across all stakeholders, with symptom tracking identified as the most universally desired feature. Despite this interest, current use of digital tools remains limited, revealing a gap between interest and adoption. Barriers such as usability, cost, digital literacy, and trust in technology may be constraining adoption. Secondary priorities differed, with people with PD and at-risk individuals favoring actionable lifestyle recommendations, caregivers desiring improved communication, and HCPs prioritizing patient and caregiver engagement. There was a statistically significant difference between persons with PD and HCPs and the desire for an AI feature that enhances patient and caregiver engagement. These findings highlight the importance of user-centered design approaches that foreground stakeholder needs and priorities, thereby enhancing the relevance, usability, and uptake of digital tools. By translating predictive insights into actionable support for self-care and collaborative decision-making, such approaches may foster sustained engagement, adherence, and more effective integration of AI-based technologies into PD care.

## Supplementary material

10.2196/90377 Multimedia Appendix 1 Overview of the survey questions included in the analysis. For exact wording, refer to [21]. HCP: health care provider; ICG: informal caregiver; PD: Parkinson disease.

10.2196/90377 Multimedia Appendix 2 Distribution of respondents.

10.2196/90377 Multimedia Appendix 3 Statistical analysis.

10.2196/90377 Checklist 1 STROBE statement—checklist of items that should be included in reports of observational studies.

## Abbreviations

AI: artificial intelligence
CHERRIES: Checklist for Reporting Results of Internet E-Surveys
HCPs: health care providers
PD: Parkinson disease
STROBE: Strengthening the Reporting of Observational Studies in Epidemiology

## References

[1] Zaman MS, Ghahari S, McColl MA (2021). Barriers to accessing healthcare services for people with Parkinson’s disease: a scoping review. J Parkinsons Dis.

[2] Seshadri S, Contento A, Sugiura K, Abendroth M, Macchi Z, Kluger BM (2024). Parkinson’s disease carepartners’ perceptions of the challenges and rewards of caregiving. Am J Hosp Palliat Care.

[3] Longacre ML, Roche L, Kueppers GC, Buurman B (2025). Parkinson’s disease and caregiving roles, demands, and support needs and experiences: a scoping review. Healthcare (Basel).

[4] Salles P, Tirapegui JM, Chaná-Cuevas P (2024). Genetics of Parkinson’s disease: dominant forms and GBA. Neurol Perspect.

[5] Su D, Cui Y, He C (2025). Projections for prevalence of Parkinson’s disease and its driving factors in 195 countries and territories to 2050: modelling study of Global Burden of Disease Study 2021. BMJ.

[6] Balestrino R, Schapira AHV (2020). Parkinson disease. Eur J Neurol.

[7] Lee NK, Kim JS (2024). Status and trends of the digital healthcare industry. Healthc Inform Res.

[8] Madanian S, Nakarada-Kordic I, Reay S, Chetty T (2023). Patients’ perspectives on digital health tools. PEC Innov.

[9] Luckhaus JL, Clareborn A, Hägglund M, Riggare S (2024). Balancing feeling 'prepared' without feeling 'devoured': a qualitative study of self-care from the perspective of self-empowered persons living with Parkinson’s disease in Sweden. Health Expect.

[10] Lee J, Yeom I, Chung ML, Kim Y, Yoo S, Kim E (2022). Use of mobile apps for self-care in people with Parkinson disease: systematic review. JMIR mHealth uHealth.

[11] AI-PROGNOSIS.

[12] von Elm E, Altman DG, Egger M (2007). The Strengthening the Reporting of Observational Studies in Epidemiology (STROBE) statement: guidelines for reporting observational studies. Epidemiology.

[13] Eysenbach G (2004). Improving the quality of Web surveys: the Checklist for Reporting Results of Internet E-Surveys (CHERRIES). J Med Internet Res.

[14] Corda KW, Quick V, Schefske S, DeCandia J, Byrd-Bredbenner C (2010). Toward a polytheoretical framework for health behavior change. Am J Health Stud.

[15] Ajzen I (1991). The theory of planned behavior. Organ Behav Hum Decis Process.

[16] Bandura A (2001). Social cognitive theory: an agentic perspective. Annu Rev Psychol.

[17] Rosenstock IM, Strecher VJ, Becker MH (1988). Social learning theory and the Health Belief Model. Health Educ Q.

[18] Davis FD (1989). Perceived usefulness, perceived ease of use, and user acceptance of information technology. MIS Q.

[19] EUSurvey.

[20] (2010). WHODAS 2.0 translation package (version 1.0): translation and linguistic evaluation protocol and supporting material. https://terrance.who.int/mediacentre/data/WHODAS/Guidelines/WHODAS%202.0%20Translation%20guidelines.pdf.

[21] Yates F (1934). Contingency tables involving small numbers and the χ2 test. Suppl J R Stat Soc Ser B.

[22] Greenhalgh T, Wherton J, Papoutsi C (2017). Beyond adoption: a new framework for theorizing and evaluating nonadoption, abandonment, and challenges to the scale-up, spread, and sustainability of health and care technologies. J Med Internet Res.

[23] Hirczy S, Zabetian C, Lin YH (2024). The current state of wearable device use in Parkinson’s disease: a survey of individuals with Parkinson’s. Front Digit Health.

[24] Hepburn J, Williams L, McCann L (2025). Barriers and facilitators to digital health technology adoption by older adults with chronic disease: an updated systematic review. Br J Gen Pract.

[25] Wannheden C, Revenäs Å (2020). How people with Parkinson’s disease and health care professionals wish to partner in care using eHealth: co-design study. J Med Internet Res.

[26] Bertolazzi A, Quaglia V, Bongelli R (2024). Barriers and facilitators to health technology adoption by older adults with chronic diseases: an integrative systematic review. BMC Public Health.

[27] Luckhaus JL, Scott Duncan T, Kharko A (2026). A qualitative exploration of ethical aspects of using AI in Parkinson disease: patient panel study. JMIR AI.

[28] Schaeffer E, Toedt I, Köhler S, Rogge A, Berg D (2021). Risk disclosure in prodromal Parkinson’s disease. Mov Disord.

[29] Schaeffer E, Rogge A, Nieding K (2020). Patients’ views on the ethical challenges of early Parkinson disease detection. Neurology (ECronicon).

[30] Paccoud I, Valero MM, Marín LC (2024). Patient perspectives on the use of digital medical devices and health data for AI-driven personalised medicine in Parkinson’s Disease. Front Neurol.

[31] Godoy Junior CA, Mäkitie L, Fiorenzato E (2025). Diverse preferences, different solutions: exploring remote monitoring preferences in Parkinson’s disease through a discrete choice experiment. J Parkinsons Dis.

[32] Ashimwe A, Davoody N (2024). Exploring health care professionals’ perspectives on the use of a medication and care support system and recommendations for designing a similar tool for family caregivers: interview study among health care professionals. JMIR Med Inform.

[33] Sun WJ, Peng YJ, Liang Y (2023). Barriers and facilitators for healthcare providers to implement family-centered care in Parkinson’s disease: a scoping review. Front Neurol.

[34] Mahmood A, Kim H, Chang CF, Kedia S, Arshad H, Dillon PJ (2024). mHealth apps use and their associations with healthcare decision-making and health communication among informal caregivers: evidence from the National Cancer Institute’s health information national trends survey. Am J Health Promot.

[35] Tolosa E, Garrido A, Scholz SW, Poewe W (2021). Challenges in the diagnosis of Parkinson’s disease. Lancet Neurol.

[36] Räty V, Kuusimäki T, Majuri J (2025). Stability and accuracy of a diagnosis of Parkinson disease over 10 years. Neurology.

[37] de Laat B, Hoye J, Stanley G (2024). Intense exercise increases dopamine transporter and neuromelanin concentrations in the substantia nigra in Parkinson’s disease. NPJ Parkinsons Dis.

