# Supplementary figures and images for: Comparing Stakeholders’ Perspectives on Parkinson Disease Management and Digital Technologies: Exploratory International Survey
Source: JMIR Form Res. 2026 May 20;10:e90377. doi: 10.2196/90377 (PMC13189255; doi:10.2196/90377)

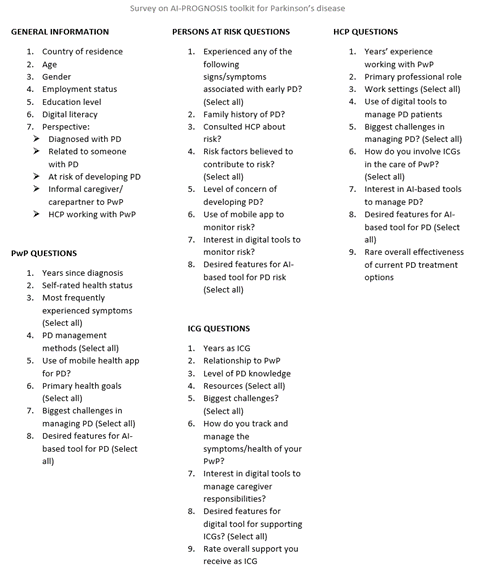

Supplement: Multimedia Appendix 1 [file formative-v10-e90377-s001.png]

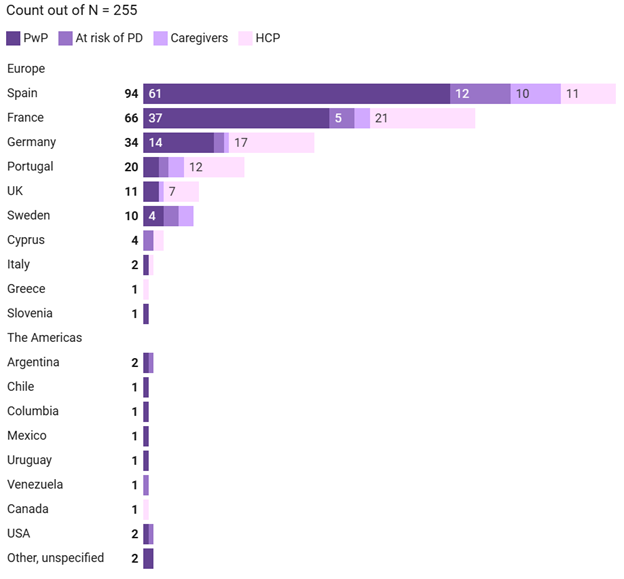

Supplement: Multimedia Appendix 2 [file formative-v10-e90377-s002.png]
