# Supplementary material for: Comparing Stakeholders’ Perspectives on Parkinson Disease Management and Digital Technologies: Exploratory International Survey
Source: JMIR Form Res. 2026 May 20;10:e90377. doi: 10.2196/90377 (PMC13189255; doi:10.2196/90377)
Supplement: Multimedia Appendix 3 [file formative-v10-e90377-s003.pdf]

Early diagnosis and risk assessment

Contingency Tables

stakeholder		Early diagnosis and risk assessment: selected	not selected	Total
PwP	Count	55.00	77.00	132.00
	Expected count	53.44	78.56	132.00
HCP	Count	28.00	45.00	73.00
	Expected count	29.56	43.44	73.00
Total	Count	83.00	122.00	205.00
	Expected count	83.00	122.00	205.00

Chi-Squared Tests

	Value	df	p
X^2	0.21	1	0.64
N	205		

Nominal

	Value
Phi-coefficient	0.03
Cramer's V	0.03

Monitoring and tracking symptoms

Contingency Tables

Stakeholder		Monitoring and tracking symptoms		
		selected	not selected	Total
pwp	Count	108.00	24.00	132.00
	Expected count	104.31	27.69	132.00
hcp	Count	54.00	19.00	73.00
	Expected count	57.69	15.31	73.00
Total	Count	162.00	43.00	205.00
	Expected count	162.00	43.00	205.00

Chi-Squared Tests

	Value	df	p
X^2	1.75	1	0.19
N	205		

Nominal

	Value
Phi-coefficient	0.09
Cramer's V	0.09

Personalized treatment recommendations

Contingency Tables

stakeholder		Personalized treatment recommendations: selected	not selected	Total
PwP	Count	101.00	31.00	132.00
	Expected count	95.94	36.06	132.00
HCP	Count	48.00	25.00	73.00
	Expected count	53.06	19.94	73.00
Total	Count	149.00	56.00	205.00
	Expected count	149.00	56.00	205.00

Chi-Squared Tests

	Value	df	p
X^2	2.74	1	0.10
N	205		

Nominal

	Value
Phi-coefficient	0.12
Cramer's V	0.12

Predicting disease progression

Contingency Tables

stakeholder		Predicting disease progression: selected	Predicting disease progression: not selected	Total
PwP	Count	63.00	69.00	132.00
	Expected count	62.46	69.54	132.00
HCP	Count	34.00	39.00	73.00
	Expected count	34.54	38.46	73.00
Total	Count	97.00	108.00	205.00
	Expected count	97.00	108.00	205.00

Chi-Squared Tests

	Value	df	p
X^2	0.03	1	0.87
N	205		

Nominal

	Value
Phi-coefficient	0.01
Cramer's V	0.01

Enhancing patient and caregiver engagement

Contingency Tables

stakeholder		enhancing: selected	enhancing: not selected	Total
PwP	Count	30.00	102.00	132.00
	Expected count	49.58	82.42	132.00
HCP	Count	47.00	26.00	73.00
	Expected count	27.42	45.58	73.00
Total	Count	77.00	128.00	205.00
	Expected count	77.00	128.00	205.00

Chi-Squared Tests

	Value	df	p
X^2	34.78	1	< .001
N	205		

Nominal

	Value
Phi-coefficient	−0.41
Cramer's V	0.41
