# Supplementary material for: Comparing Stakeholders’ Perspectives on Parkinson Disease Management and Digital Technologies: Exploratory International Survey
Source: JMIR Form Res. 2026 May 20;10:e90377. doi: 10.2196/90377 (PMC13189255; doi:10.2196/90377)
Supplement: Checklist 1 [file formative-v10-e90377-s004.pdf]

STROBE Statement—checklist of items that should be included in reports of observational studies

	Item No.	Recommendation	Page No.	Relevant text from manuscript
**Title and abstract**	1	(*a*) Indicate the study's design with a commonly used term in the title or the abstract	2	Title: Comparing Stakeholders' Perspectives on Parkinson's Disease Management and Digital Technologies: An Exploratory International Survey<br><br>Abstract: "An anonymous cross-sectional online survey of exploratory nature was distributed (from Dec. 2024 to Oct. 2025) in five languages"
		(*b*) Provide in the abstract an informative and balanced summary of what was done and what was found	2	The results, a summary of the findings, and brief discussion of the implications are presented in the abstract.
**Introduction**				
Background/rationale	2	Explain the scientific background and rationale for the investigation being reported	3	"A scoping review on mHealth tools for PD management found that the majority of tools support clinical symptom assessment, but there is a need for more tools that facilitate self-care through symptom management [10].10 Additionally, predictive AI is a novel and promising addition to PD management, though AI likely requires mobile apps and/or smartwatches to collect data, which distinguish it from traditional self-management systems, warranting further research. One such initiative is AI-PROGNOSIS [11],11 an EU-funded project that aims to improve PD diagnosis, monitoring and treatment through predictive AI. To ensure user-centered design, it is critical to understand the current experiences, expectations, and digital readiness of the diverse groups involved in PD care. However, despite growing interest in AI for PD, user priorities and perspectives on these tools remain unclear, especially across different stakeholders."
Objectives	3	State specific objectives, including any prespecified hypotheses	3-4	Building on the identified gaps in understanding stakeholder perspectives, this study pursues four interconnected aims:<br><br>1. to identify current digital practices for PD management among PwP, persons at risk of PD, caregivers, and HCPs,

				2. to compare similarities and differences in these practices across stakeholder groups,<br>3. to explore stakeholder-specific desires and expectations for AI-based tools, including predictive and preventive applications, and<br>4. to assess how these practices and preferences align or diverge, thereby highlighting unmet needs and opportunities to inform the tailored design of future AI-driven solutions for PD care.
**Methods**				
Study design	4	Present key elements of study design early in the paper	4	"The present study is reported in accordance with the STROBE checklist for cross-sectional studies [12] (see Appendix 1) and the Checklist for Reporting Results of Internet E-Surveys (CHERRIES) [13]."<br>"The survey was conducted within the scope of the EU-funded AI-PROGNOSIS project…"
Setting	5	Describe the setting, locations, and relevant dates, including periods of recruitment, exposure, follow-up, and data collection	5	"Data was collected between December 2024 and October 2025."
Participants	6	*Cross-sectional study*—Give the eligibility criteria, and the sources and methods of selection of participants	5	"The online survey consisted of 21 to 23 questions, with the exact number depending on the respondent's group, i.e, PwP, persons at risk of PD, caregivers, and HCPs."<br>"Survey dissemination was done through the clinical and patient networks of the AI-PROGNOSIS project partners, focusing on European countries where the languages available to the research team are spoken (the UK, France, Germany, Spain, and Sweden). However, no geographical restrictions were placed, so responses from all countries were possible."<br>"A non-probability sampling was used as an exploratory sample with no random selection. Inclusion criteria for respondents were over 18 years of age and self-identified as one of the following: (1) having a Parkinson's diagnosis, (2) being at risk of PD, (3) being related to someone with PD, (4) being a caregiver to PwP, or (5) current healthcare provider who works with PwPs."

Variables	7	Clearly define all outcomes, exposures, predictors, potential confounders, and effect modifiers. Give diagnostic criteria, if applicable	5	**Outcomes** were defined as responses to the survey items assessing perceptions of digital health tools and predictive AI for PD, as specified in the Methods section.<br><br>**Exposures and predictors** included participant role (self-identified individual with PD, healthcare professional working with PD patients, self-identified as at risk for PD or being related to someone with PD, or as being a caregiver to someone with PD), demographic characteristics, and other self-reported variables collected via the survey.<br><br>**Potential confounders** were not adjusted for, as the study was descriptive in nature and analyses were limited to unadjusted, exploratory comparisons using chi-square tests.<br><br>**Effect modifiers** were not formally assessed.<br><br>**Diagnostic criteria** were not applied, as Parkinson's disease status was based on self-identification rather than clinical diagnosis.
Data sources/ measurement	8*	For each variable of interest, give sources of data and details of methods of assessment (measurement). Describe comparability of assessment methods if there is more than one group	5	Data for all variables were obtained through a self-administered survey. Outcomes and predictors were assessed using self-reported responses to survey items. Participant role was determined based on participant self-classification.
Bias	9	Describe any efforts to address potential sources of bias	5	Potential sources of bias include self-selection and self-report bias. No specific methods were used to mitigate these biases, as the study was descriptive in nature and based on voluntary survey participation.
Study size	10	Explain how the study size was arrived at		The survey was exploratory in nature; therefore, no formal sample size or power calculation was performed. Study size was determined by the number of participants who responded during the survey period. "In this paper, only Part 1: General information (41 items between all stakeholder versions) was

	analyzed (Figure 1), which was intended to provide an exploratory snapshot…”

Continued on next page

Quantitative variables	11	Explain how quantitative variables were handled in the analyses. If applicable, describe which groupings were chosen and why		Quantitative variables were not analyzed as continuous measures. Survey responses and participant characteristics were analyzed as categorical variables as collected. No additional grouping or transformation of quantitative variables was performed. See "analysis" section.
Statistical methods	12	(*a*) Describe all statistical methods, including those used to control for confounding		"These multiple-choice questions were analyzed descriptively, summarized through frequency tables, figures, and in-text descriptions. Similar responses (e.g., "good" and "excellent") were dichotomized, where applicable. Analysis was carried out by JLL in JASP v0.19.1.0. Where the same question was asked of multiple stakeholder groups, these responses are presented side-by-side in figures or text for comparison. Chi-square tests were performed to examine stakeholder differences in desired AI features. Because all comparisons involved 2×2 contingency tables, Yates' continuity correction was applied [21]. Open-ended qualitative questions were not included due to low response rates."
		(*b*) Describe any methods used to examine subgroups and interactions		"Chi-square tests were performed to examine stakeholder differences in desired AI features."
		(*c*) Explain how missing data were addressed	4	N/A: All analyzed survey questions were mandatory. "The online survey consisted of 21 to 23 questions, with the exact number depending on the respondent's group, i.e, PwP, persons at risk of PD, caregivers, and HCPs. Some questions asked respondents to rate statements. Including the latter, the survey consists of 48 items. 15-17 items were mandatory (the number of questions depended on the stakeholder group) closed single-choice and multiple-choice questions, 48 mandatory 5-point Likert-scale items (ranging from 1 — strongly disagree through 3 — Neutral to 5 — Strongly agree), and six optional free-text comments."
		(*d*) *Cross-sectional study*—If applicable, describe analytical methods taking account of sampling strategy	5	"A non-probability sampling was used…"
		(*e*) Describe any sensitivity analyses		N/A
**Results**				
Participants	13*	(a) Report numbers of individuals at each stage of study—eg numbers potentially eligible, examined for		

		eligibility, confirmed eligible, included in the study, completing follow-up, and analysed		
		(b) Give reasons for non-participation at each stage		
		(c) Consider use of a flow diagram		
Descriptive data	14*	(a) Give characteristics of study participants (eg demographic, clinical, social) and information on exposures and potential confounders	7	See "respondent characteristics" and Table 1.
		(b) Indicate number of participants with missing data for each variable of interest		N/A: All analyzed survey questions were mandatory.
Outcome data	15*	*Cohort study*—Report numbers of outcome events or summary measures over time		N/A: not a cohort study
		Case-control study—Report numbers in each exposure category, or summary measures of exposure		N/A: not a case-control study
		Cross-sectional study—Report numbers of outcome events or summary measures	7-12	See Results section (p. 7-12)
Main results	16	(*a*) Give unadjusted estimates and, if applicable, confounder-adjusted estimates and their precision (eg, 95% confidence interval). Make clear which confounders were adjusted for and why they were included		N/A
		(*b*) Report category boundaries when continuous variables were categorized		N/A: no continuous variables were categorized.
		(*c*) If relevant, consider translating estimates of relative risk into absolute risk for a meaningful time period		N/A

Continued on next page

Other analyses	17	Report other analyses done—eg analyses of subgroups and interactions, and sensitivity analyses		N/A
**Discussion**				
Key results	18	Summarise key results with reference to study objectives	13	"This study explored how different stakeholder groups, PwP, individuals people at risk, informal caregivers, and healthcare professionals, currently engage with digital tools, what they expect from AI-based solutions, and where their priorities converge or diverge in managing Parkinson's disease. The results highlight the complexity of managing PD and suggest both universal needs and distinct priorities across these four groups regarding the potential application of predictive AI. Interest in predictive AI was high across all stakeholder groups, with symptom tracking being the most desired feature; however, secondary priorities varied by group. Additionally, despite this interest, reported technological use was limited. Most PwP did not use symptom-tracking apps or wearables nor currently track their condition, though many expressed intentions to start. This discrepancy suggests that barriers such as usability, cost, digital literacy, and trust in technology may be constraining adoption. This pattern aligns with broader digital health literature demonstrating that interest in technology does not necessarily translate into adoption or sustained use [22]. Prior studies have found cost [23], discomfort (of wearables) [23], and fear of replacing in-person care [24] to constrain adoption for older adults with chronic illness. Addressing these barriers will be essential if AI-driven solutions are to move from intention to sustained practice."
Limitations	19	Discuss limitations of the study, taking into account sources of potential bias or imprecision. Discuss both direction and magnitude of any potential bias	16	Strengths and limitations: "The present study employed a non-probability sampling approach, with recruitment primarily conducted through project networks and online dissemination channels. This strategy enabled participation from multiple countries and stakeholder groups, enhancing the overall diversity and breadth of perspectives captured. However, this same approach may have introduced sampling biases, including uneven representation across stakeholder groups and an overrepresentation of

				highly educated respondents, with 80% holding a bachelor's degree or higher. Combined with the relatively small sample size and exploratory nature of the study, these factors limit the generalizability of subgroup comparisons. Accordingly, the findings should be interpreted as preliminary and intended to inform future large-scale studies using more systematic and inclusive recruitment strategies.<br>While multi-country inclusion is a strength, it also introduces challenges in interpretation. Contextual factors such as healthcare systems, access to digital tools, and cultural attitudes toward technology vary widely across the countries included. These wide contextual differences may have influenced how participants perceived and responded to questions about symptom management and digital support tools. Additionally, although the AI tools were briefly explained (including functions, features, aims), differing understanding of AI likely influenced responses and acceptability."
Interpretation	20	Give a cautious overall interpretation of results considering objectives, limitations, multiplicity of analyses, results from similar studies, and other relevant evidence	12-18	See the Discussion section.
Generalisability	21	Discuss the generalisability (external validity) of the study results	16	"Combined with the relatively small sample size and exploratory nature of the study, these factors limit the **generalizability** of subgroup comparisons. Accordingly, the findings should be interpreted as preliminary and intended to inform future large-scale studies using more systematic and inclusive recruitment strategies."
**Other information**				
Funding	22	Give the source of funding and the role of the funders for the present study and, if applicable, for the original study on which the present article is based	18	"This research is part of the AI-PROGNOSIS project funded by the European Union (EU) under Grant Agreement No. 101080581. Views and opinions expressed are, however, those of the authors, and do not necessarily reflect those of the EU or European Health and Digital Executive Agency (EHDEA). Neither the EU nor the EHDEA can be held responsible for them."

*Give information separately for cases and controls in case-control studies and, if applicable, for exposed and unexposed groups in cohort and cross-sectional studies.

Note: An Explanation and Elaboration article discusses each checklist item and gives methodological background and published examples of transparent reporting. The STROBE checklist is best used in conjunction with this article (freely available on the Web sites of PLoS Medicine at http://www.plosmedicine.org/, Annals of Internal Medicine at http://www.annals.org/, and Epidemiology at http://www.epidem.com/). Information on the STROBE Initiative is available at www.strobe-statement.org.
